# Keratinocyte Growth Factor-2 Reduces Inflammatory Response to Acute Lung Injury Induced by Oleic Acid in Rats by Regulating Key Proteins of the Wnt/*β*-Catenin Signaling Pathway

**DOI:** 10.1155/2020/8350579

**Published:** 2020-06-20

**Authors:** Shao Tenghao, Chen Ning, Wang Shenghai, Sun Qinlong, Wu Jiaqian, Wang Kuo, Yu Zhanbiao, Ma Xigang

**Affiliations:** ^1^Department of Critical Care Medicine, Affiliated Hospital of Hebei University, Baoding, Hebei 071000, China; ^2^Department of Critical Care Medicine, General Hospital of Ningxia Medical University, Yinchuan, Ningxia 750004, China

## Abstract

Reducing inflammation can effectively relieve acute lung injury (ALI). *Objective*. To test whether keratinocyte growth factor-2 (KGF-2) can reduce oleic acid-induced inflammation in ALI of rats and explore its possible mechanism. *Methods*. 45 Sprague-Dawley rats were randomly divided into control group, ALI group, and ALI + KGF-2 group. The animal model of acute lung injury was established by injecting 0.1 mL/kg oleic acid into the tail vein of rats. Rats in the control group were injected with equal volume of normal saline (NS). Each group needs pretreatment 72 hours before the preparation of the acute lung injury model. The control group and ALI group were instilled with 5 ml/kg NS through the airway, and the same amount of KGF-2 was instilled in the ALI + KGF-2 group. It takes 8 hours to successfully prepare the ALI model. Observe the pathological changes of lung tissue through light microscopy, ultrastructural changes through electron microscopy, and the lung wettability/dry weight (w/d) ratio and lung permeability index (LPI). By detecting changes in inflammatory factors in lung tissue and changes in the number of BALF cells, the changes in inflammation in each group were observed. The expressions of Wnt5a, *β*-catenin, and APC in lung tissue were detected by immunohistochemistry and Western blot. The changes of key proteins in Wnt/*β*-catenin signaling pathway in the lung tissue of each group were observed. *Result*. Compared with the ALI group, after KGF-2 pretreatment, the degree of lung injury was reduced, the expression of inflammatory factors was reduced, and the number of red blood cells and white blood cells in BALF was reduced. It can also be observed that the expression of Wnt5a, *β*-catenin, and APC, a key protein in the Wnt/*β*-catenin signaling pathway, is reduced. The analysis showed that the number of inflammatory factors, red blood cells, and white blood cells in BALF was positively correlated with the expression of Wnt5a, *β*-catenin, and APC. *Conclusion*. KGF-2 may reduce the inflammatory response in ALI induced by oleic acid by regulating key proteins in the Wnt/*β*-catenin signaling pathway.

## 1. Introduction

The loss of control and the outbreak of inflammatory response are the basis of the onset of ALI [1, 2]. A series of factors such as infection, multiple injuries, and shock can destroy the body's defense structure, release a large number of white blood cells and inflammatory mediators, and directly destroy the alveolar-capillary barrier, eventually leading to high permeability alveolar edema and transparent membrane formation [3, 4]. Insufficient inflammatory response is not enough to clear the pathogen, and excessive inflammatory response will cause tissue damage, so it is particularly important to seek the control point of the inflammatory response. Wnt/*β*-catenin signaling pathway is closely related to inflammatory diseases [5, 6]. Activation of the Wnt/*β*-catenin signaling pathway is found in various inflammation-related lung diseases [7]. Wnt5a, *β*-catenin, and APC are key proteins in the Wnt5a/*β*-catenin classic signaling pathway and play an important role in the study of the entire pathway. The Wnt5a/*β*-catenin classic signaling pathway is one of the Wnt/*β*-catenin signaling pathways. Keratinocyte growth factor-2 (KGF-2) is secreted by fibroblasts and other mesenchymal cells. It can be combined with fibroblast growth factor receptor 2IIIb (FGFR2IIIb) expressed in epithelial cells to promote epithelial cell proliferation [8–11]. However, the regulatory effect of KGF-2 on inflammation and its specific mechanism are not fully understood. Therefore, the first step of this study was to observe the protective effect of KGF-2 on ALI rats. The second step is to observe whether it has a regulatory effect on inflammation. The third step is to detect whether KGF-2 has a regulatory effect on key proteins in the Wnt/*β*-catenin signaling pathway. Initially, the mechanism of action of KGF-2 on ALI is explored.

## 2. Materials and Methods

### 2.1. Animals

Forty-five Sprague-Dawley rats of about 220 ± 20 g were kept in a clean, temperature-controlled, and independent ventilation environment. All SD rats were free to obtain food and water. All experimental protocols were approved by the ethics committee.

### 2.2. Grouping and Animal Handling

Forty-five SD rats were randomly divided into three groups: control group, ALI group, and ALI + KGF-2 group. The preparation of the rat ALI model was completed by injecting 0.1 mL/kg oleic acid (Sigma, USA) into the tail vein of the rat. The control group was injected with 0.1 mL/kg saline (NS) in the tail vein as a control. Three groups were instilled by tracheal infusion 72 h before the tail vein injection. The ALI + KGF-2 group was infused with 5 mg/kg of KGF-2, and the control group and ALI group were infused with 5 mg/kg of NS.

### 2.3. Lung Morphometry Analyses

After the rat ALI model was successfully prepared, the lung tissue was quickly put into 4% paraformaldehyde to fully fix it, and then the paraformaldehyde on the surface of the lung tissue was washed away with clean water. Different concentrations of alcohol were placed in the lung tissue in turn, and then the lung tissue was placed in xylene. Finally, it was embedded in paraffin to form a wax block. Continuously it was cut into 4 *μ*m thick slices, hematoxylin staining to take the nucleus, eosin staining to take the cytoplasm, and taking pictures under an optical microscope. Five fields of vision were taken from each slice for ALI pathology scoring [12]. One point is thickening of the alveolar interval, one point is alveolar hemorrhage, one point is fibrin deposition in the alveoli, and one point is infiltration of inflammatory cells in the alveoli. Finally, we calculated the average of 5 visual fields as the lung injury score (LIS) of the slice [13].

### 2.4. Transmission Electron Microscope

NS cleared the blood stains on the surface of lung tissue and took the rat lung tissue into 1% osmium tetroxide for fixation. Different concentrations of alcohol were placed in the lung tissue in turn and embedded in propylene oxide to form a wax block. After cutting into thin slices, they were dyed with uranyl acetate and lead citrate, respectively. The ultrastructure of rat lung tissue was observed under transmission electron microscope.

### 2.5. Lung Wet-to-Dry Weight Ratio (W/D)

Open the rat chest cavity and ligate the right lung. Remove the upper right lung tissue, remove water and blood stains with gauze, and weigh the lung tissue as the wet weight. Place the lung tissue in a drying cabinet at 80°C for 72 h, and weigh it as the dry weight. The wet weight/dry weight is wet-to-dry weight ratio (W/D).

### 2.6. Lung Permeability Index (LPI)

Connect the catheter to the trachea. Inject 2 ml NS slowly into the catheter to get the NS into the lungs for 5 times. Then slowly withdraw NS. Collect approximately 7 ml of bronchoalveolar lavage fluid (BALF). Blood was collected through the abdominal aorta of rats. Centrifuge at 3000 r/min for 15 minutes to collect blood and BALF supernatant. BCA protein assay was used to detect the protein concentration in plasma and BALF. The protein concentration in BALF/plasma protein concentration is LPI.

### 2.7. Enzyme-Linked Immunosorbent Assay (ELISA)

Take an ELISA kit (Nanjing Senbega Biotechnology Co., Ltd., Nanjing, China), set up standard wells, and establish a standard curve. Set up sample wells, and add sample and horseradish peroxidase (HRP) labeled secondary antibody to each well in turn. After incubating in a 37°C incubator for 30 minutes, each well was washed 3 times. Add substrate and stop solution to each well in turn. Finally, the value of each well is tested, and the concentration of each sample is determined according to the previously established standard curve.

### 2.8. Wright's Giemsa Staining

Drop BALF onto the slide, drip Wright's Giemsa A onto the smear, and cover the specimen with staining solution for 1 min. Then, drop Wright's Giemsa B onto Wright's Giemsa A to mix the two liquids. Finally, the running water rinses off the dyeing solution. Take pictures under an optical microscope. Count the number and proportion of white blood cells and red blood cells, respectively.

### 2.9. Immunohistochemistry

Lung tissue was cut into 4 *μ*m thick slices. Repair antigen under high pressure in sodium citrate (pH = 6.0). Wnt5a antibody (1 : 100; Abcam; Cambridge, MA, USA), *β*-catenin antibody (1 : 500; Abcam; Cambridge, MA, USA), and APC rabbit antibodies (1 : 100; Abcam; Cambridge, MA, USA) were incubated overnight at 4°C and negative controls were incubated with PBS. On the next day, horseradish peroxidase (HRP) labeled goat anti-rabbit IgG (Beijing Zhongshan Jinqiao Technology Co., Ltd.) was added dropwise. The incubation temperature was 37°C for 30 minutes and rinsed with PBS three times. Add 3,3′-diaminobenzidine (DAB) (Beijing Zhongshan Jinqiao Technology Co., Ltd.) for color development. Hematoxylin was counterstained and mounted. Observed under light microscope, the tissues were dark brown for protein positive expression. No coloration or light coloration is negative or weak positive expression. Finally, the average optical density (AOD) expressed by IPP software was analyzed. The integrated optical density (IOD)/positive expression area is AOD.

### 2.10. Western Blot Analyses

The tissue is quickly removed from the liquid nitrogen, and the lung tissue is ground into a powder. Collect lung tissue powder into a centrifuge tube, and add tissue lysate and pancreatin, respectively. The supernatant after centrifugation is a protein extract. BCA protein assay was used to determine protein concentration. Protein separation is done by polyacrylamide gel electrophoresis. The electrophoretic transfer method of wet transfer is used to transfer the protein to the PVDF membrane and block it in skim milk. Wnt5a antibody (1 : 500; Abcam; Cambridge, MA, USA), *β*-catenin antibody (1 : 5000; Abcam; Cambridge, MA, USA), APC antibody (1 : 500; Abcam; Cambridge, MA, USA), and GAPDH antibody (1 : 500; Abcam; Cambridge, MA, USA) were incubated overnight at 4°C. Wash the PVDF membrane 3 times with Tris-HCl buffered saline + Tween (TBST). A secondary antibody labeled with horseradish peroxidase (goat anti-rabbit IgG) was added for reaction. TBST washed the PVDF membrane 3 times again. Finally, with the help of ECL chemiluminescent reagents, the PVDF film shows the image. Analysis was done using Image J software.

### 2.11. Statistical Analysis

All measured data are expressed as mean ± standard deviation. One-way analysis of variance was used to detect differences in normal distribution data between groups, and the LSD test was used for comparison between groups. One-way analysis of variance is used after variable transformation of data with uneven variance. After nonnormal distribution data is subjected to rank transformation, nonparametric test is used. For bivariate normal distribution data, straight-line correlation analysis is used, and for nonlinear trend data, curve fitting analysis is used. All statistical analysis was completed using statistical software SPSS23.0; *P* < 0.05 was considered statistically significant.

## 3. Results

### 3.1. KGF-2 Has a Protective Effect on the Lung Tissue of ALI Rats Induced by Oleic Acid

**Figure 1:**
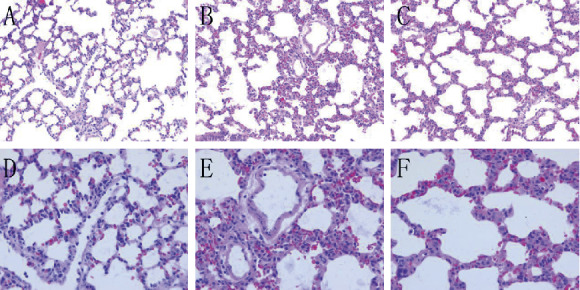
(a) The alveoli of the control group were normal lung tissue structure (200x). (b) Alveolar septa thickened and fusion changes occurred in the ALI group (200x). (c) Alveoli in the ALI + KGF-2 group were slightly fused and the alveolar interval was slightly thickened (200x). (d) The alveolar of the control group is normal lung tissue structure (400x). (e) A large number of inflammatory cells infiltrate in the ALI group, and the lung interstitium is obviously congested (400x). (f) A small amount of inflammatory cell infiltration in the ALI + KGF-2 group and a small amount of congestion in the lung interstitium (400x).

Hematoxylin-eosin staining was used to observe the pathological changes (Figure 1). The control group had normal lung tissue structure, with complete and clear alveolar structures (Figures 1(a) and 1(d)). In the ALI group, the alveolar interval of the lung tissue was thickened, the alveolar space was reduced, a large number of microthrombi formed in the blood vessels, and a large number of inflammatory cells accumulated in the alveolar cavity and the pulmonary interstitial hyperemia (Figures 1(b) and 1(e)). Pulmonary congestion and edema were alleviated in the ALI + KGF-2 group, and a small amount of inflammatory cells infiltrated the interstitial lung (Figures 1(c) and 1(f)). Compared with the control group, the LIS value of the ALI group increased significantly (*P* < 0.01). Compared with the ALI group, the LIS in the ALI + KGF-2 group was reduced (*P* < 0.01) but was higher than the control group (*P* < 0.01) (Table 1).

**Figure 2:**
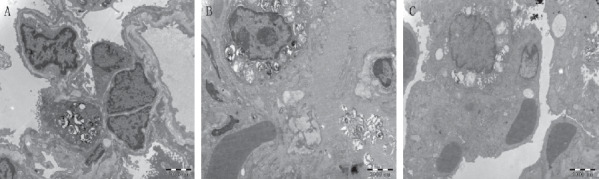
Ultrastructural changes of lung tissue of rats in each group under transmission electron microscope (10,000 times). (a) The alveolar-capillary barrier of the control group is intact. (b) The alveolar-capillary barrier in the ALI group was severely damaged, the alveolar type II epithelial cells were degenerated, the endothelial cells were apoptosis, and the basement membrane was destroyed. (c) The damage in the ALI + KGF-2 group was alleviated, the alveolar-capillary barrier was basically complete, and a small amount of osmium lamellar bodies and mitochondria were vacuolated.

Transmission electron microscopy observed ultrastructural changes (Figure 2). The control group showed intact pulmonary microvascular endothelial cells, alveolar type I epithelial cells, and alveolar type II epithelial cells. The cell structure is regular, the cell nucleus is obvious, and the cytoplasm is uniform (Figure 2(a)). Compared with the control group, in the ALI group, the cell structure was disordered, the basement membrane structure was completely destroyed, and pulmonary microvascular endothelial cells were apoptosis and necrosis. Alveolar type I epithelial cells and alveolar type II epithelial cells have different degrees of degeneration and destruction, osmium lamellar bodies and mitochondria have varying degrees of vacuolation, and a large number of red blood cells accumulate in microvessels (Figure 2(b)). Compared with the ALI group, the morphology of alveolar type I epithelial cells and alveolar type II epithelial cells in the ALI + KGF-2 group were generally normal, and the osseous lamellar body and mitochondrial vacuolation were significantly reduced. The microvascular endothelial cells were slightly swollen and the basement membrane was intact (Figure 2(c)).

LPI value and w/d ratio were used to observe the changes in lung permeability of rats in each group (Table 1). Compared with the control group, the lung LPI value and w/d ratio of the ALI group increased significantly (*P* < 0.01). After KGF-2 pretreatment, the lung LPI value and w/d ratio were significantly lower than those of the ALI model group (both *P* < 0.01). Compared with the control group, the lung LPI value and w/d ratio of the ALI + KGF-2 group increased (*P* < 0.01).

### 3.2. KGF-2 Can Reduce Oleic Acid-Induced Lung Inflammation in ALI Rats

ELISA was used to detect the expression levels of TNF-*α* and IL-10 in lung inflammation (Table 2). Compared with the control group, the expression of TNF-*α* in the lung tissue of the ALI group increased significantly (*P* < 0.01), while the expression level of IL-10 decreased (*P* < 0.01). Compared with the ALI group, the TNF-*α* expression level in the lung tissue of the ALI + KGF-2 group decreased (*P* < 0.01), and the IL-10 expression increased (*P* < 0.01).

**Figure 3:**
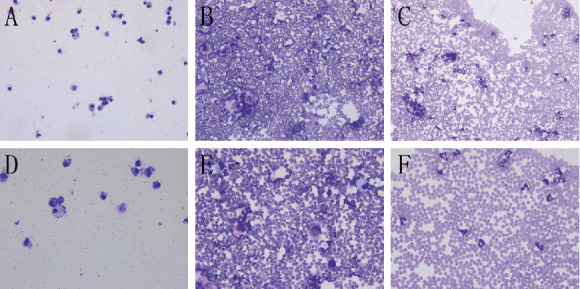
The changes in the number of cells in the BALF of each group are observed under an optical microscope. (a) White blood cells (200x) scattered in the BALF of the control group. (b) The erythrocytes in the BALF of the ALI group were full (200x). (c) The erythrocytes in the BALF of the ALI + KGF-2 group were fully covered (200x). (d) A small amount of white blood cells and no red blood cells (400x) in the BALF of the control group. (e) A large number of white blood cells (400x) can be seen in the BALF of the ALI group. (f) White blood cells (400x) are dispersed in the BALF of the ALI + KGF-2 group.

Wright's Giemsa staining was used to observe the inflammation expression in BALF (Figure 3 and Table 2). Compared with the control group (Figures 3(a) and 3(d)), the white blood cells in BALF of the ALI group increased significantly, and a large number of red blood cells were present (Figures 3(b) and 3(e)). Compared with the ALI group, the white blood cells and the number of red blood cells in the BALF of the ALI + KGF-2 group were reduced (Figures 3(c) and 3(f)). Compared with the control group, the white blood cells in the BALF of the ALI + KGF-2 group increased, and the number of red blood cells increased significantly.

### 3.3. KGF-2 Can Regulate Key Protein Changes in Wnt/*β*-Catenin Signaling Pathway

**Figure 4:**
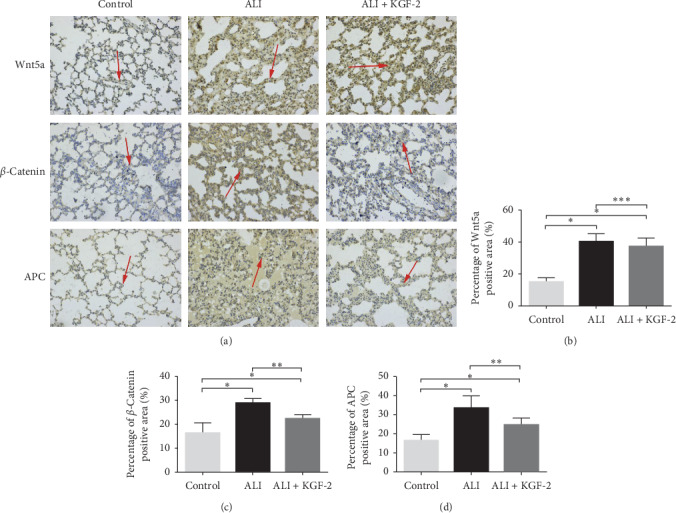
(a) Immunohistochemical staining to determine the expression of Wnt5a, *β*-catenin, and APC in rat lung tissue (×200). The brown bands with positive expression indicated by arrows (b) are statistical results ^*∗*^*P* < 0.01 versus control group; ^*∗∗*^*P* < 0.01 versus ALI group; ^*∗∗∗*^*P* > 0.05 versus ALI group).

Immunohistochemical analysis of Wnt5a, *β*-catenin, APC expression changes (Figure 4). In the control group, Wnt5a expression was weakly positive (light yellow), *β*-catenin expression was negative (light yellow), and APC expression was weakly positive (light yellow). In the ALI group, Wnt5a expression was strongly positive (dark brown), *β*-catenin expression was strongly positive (dark brown), and APC expression was strongly positive (dark brown). The lung tissue of ALI + KGF-2 group showed positive Wnt5a expression (brown yellow), *β*-catenin expression was weakly positive (light brown), and APC expression was weakly positive (light brown). Compared with the control group, the positive expressions of Wnt5a, *β*-catenin, and APC in the ALI group increased significantly (all *P* < 0.01). After KGF-2 pretreatment, the positive expression of *β*-catenin and APC in the lung tissue of rats was significantly lower than that of the ALI group (both *P* < 0.01), and there was no significant change in the positive expression of Wnt5a (*P* > 0.05).

**Figure 5:**
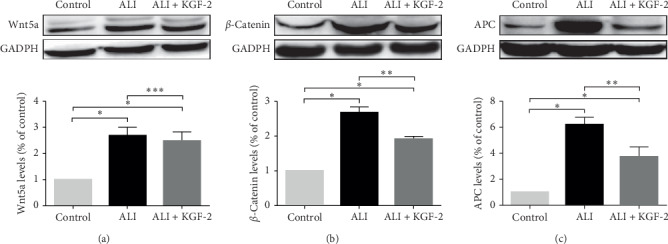
Western blot detection of Wnt5a, *β*-catenin, and APC expression in rat lung tissues. (a) Wnt5a protein polyacrylamide gel electrophoresis and protein expression level statistics. (b) *β*-Catenin protein polyacrylamide gel electrophoresis and protein expression level statistics. (c) APC protein polyacrylamide gel electrophoresis and protein expression level statistics. ^*∗*^*P* < 0.01 versus control group; ^*∗∗*^*P* < 0.01 versus ALI group; ^*∗∗∗*^*P* > 0.05 versus ALI group).

Western blot analyzed the changes of Wnt5a, *β*-catenin, and APC protein expression in the lung tissue of each group (Figure 5). Compared with the control group, the expression of Wnt5a, *β*-catenin, and APC protein in lung tissue of ALI group increased significantly (all *P* < 0.01). After KGF-2 pretreatment, the expression of Wnt5a protein in lung tissue was not significantly lower than that of ALI model group (*P* > 0.05). The expression of *β*-catenin and APC protein was lower than that of the ALI model group (*P* < 0.01), but it was still higher than that of the control group.

### 3.4. Oleic Acid-Induced Inflammation in Rat ALI Is Positively Correlated with the Expression of Wnt/*β*-Catenin Signaling Pathway Key Protein (Table 3)

In the ALI group, Wnt5a, *β*-catenin, and APC protein expression levels were positively correlated with TNF-*α* expression level and negatively correlated with IL-10 expression level. Wnt5a, *β*-catenin, and APC protein expression levels were positively correlated with the number of red blood cells and white blood cells in BALF. In ALI + KGF-2 group, *β*-catenin and APC protein expression levels were positively correlated with TNF-*α* expression level and negatively correlated with IL-10 expression level. *β*-Catenin and APC protein expression levels were positively correlated with the number of red blood cells and white blood cells in BALF. In ALI + KGF-2 group, Wnt5a protein expression level had no correlation with TNF-*α* expression level; it had a negative correlation with IL-10 expression level. There was no correlation between *β*-catenin and APC protein expression levels and the number of red blood cells and white blood cells in BALF.

## 4. Discussion

In this study, 0.1 ml/kg oleic acid was injected into the tail vein to induce ALI. After being injected with oleic acid, the rats gradually developed mental depression, increased breathing amplitude, and increased breathing frequency. After 8 hours, the thoracic cavity of the rat was exposed, a large amount of lung tissue was congested, and the lung tissue was significantly swollen. Under the light microscope, the alveolar interval was thickened, the alveolar space was reduced, and a large number of inflammatory cells accumulated in the alveolar cavity [12, 14]. To sum up, it indicates that the ALI mold making is successful.

ARDS is an acute respiratory failure caused by ALI, and its pathological feature is diffuse alveolar injury [15, 16]. This study starts from the whole experimental animals. Observation of the pathological structure changes under light microscope showed that inflammatory cells infiltrated significantly in the ALI group, the alveolar interval thickened, and the lung injury was obvious. By observing the ultrastructural changes under the electron microscope, the endothelial cells in the ALI group had apoptosis, the cell boundaries were unclear, and the whole was dissolved into one piece. It shows that the damage of alveolar-capillary barrier is obviously expressed in oleic acid-induced ALI rats. The alveolar-capillary barrier is an important structure for maintaining gas exchange in the lung tissue. When this barrier is damaged, a large amount of fluid leaks and oxygen exchange in the lung tissue is impaired [17, 18]. LPI and w/d ratio were used to observe the changes of lung permeability of rats in each group. The leaked protein and water can analyze the damage of the alveolar-capillary barrier.

In ALI, the pathogenic factor can directly act on the alveolar membrane to cause ALI, and at the same time, the pathogenic factor can directly activate white blood cells and indirectly damage the lung tissue [19–21]. After a large number of leukocytes chemoattract lung tissue, a large amount of free radicals and proteases are released to damage the lung microvascular endothelial cells [22–24]. In this study, the expression of inflammation was detected by observing changes in cells in BALF and changes in inflammatory factors in lung tissue homogenate. The erythrocyte and leukocyte counts in the ALI group increased significantly. Leukocyte exudation may be related to apoptosis of lung tissue. Leukocytes cause apoptosis of cells due to direct or indirect damage to lung microvascular endothelial cells or lung epithelial cells. A large number of red blood cells can be seen in BALF, and a large amount of red blood cell leakage indicates that the tissue has hypoxic symptoms, leading to respiratory distress. Examination of TNF-*α* and IL-10 revealed that TNF-*α* expression increased and IL-10 expression decreased in the ALI group. As a proinflammatory mediator, TNF-*α* causes severe damage to pulmonary microvascular endothelial cells and alveolar epithelial cells, causing cell apoptosis, vascular damage, and thrombosis, increasing lung tissue permeability and hypoxia [25, 26]. At the same time, TNF-*α* can promote the release of a variety of inflammatory mediators and further expand the inflammatory response, thereby aggravating lung tissue damage [27, 28]. TNF-*α* can also activate the blood coagulation system and complement system, so that the inflammation “cascades expansion” and spreads in the lung tissue [25, 28, 29]. IL-10 is an anti-inflammatory mediator, and its decrease indicates increased expression of inflammation [30, 31].

Wnt/*β*-catenin signaling pathway can not only regulate growth, value added, and differentiation, but also regulate inflammation, fibrosis, and other pathological processes [32–34]. Wnt/*β*-catenin signaling pathway includes Wnt family secreted protein (Wnt), *β*-catenin, Axin, glycogen synthase kinase 3 (GSK3), and APC protein [35–37]. Among them, Wnt5a, *β*-catenin, and APC proteins are the key proteins of Wnt5a/*β*-catenin signaling pathway. Wnt5a is an upstream protein in the classical pathway of Wnt5a/*β*-catenin signaling pathway. The Wnt5a/*β*-catenin signaling pathway is an important part of the entire Wnt/*β*-catenin signaling pathway family. Activation of Wnt protein can start the regulation of the entire Wnt/*β*-catenin signaling pathway. *β*-Catenin is the core regulator of Wnt/*β*-catenin signaling pathway, and its expression level determines the opening of the pathway [38]. APC protein is a key regulator of Wnt/*β*-catenin signaling pathway. On the one hand, APC protein is involved in the recognition, phosphorylation, and targeted degradation of *β*-catenin. On the other hand, APC protein may act as a nuclear shuttle protein and play certain roles in the nuclear transport of *β-*catenin, thereby reducing its nuclear level and transcriptional activity [39]. In this study, immunohistochemistry and Western blot were used to analyze Wnt5a, *β*-catenin, and APC in the lung tissues of each group of rats. The results showed that the expression of Wnt5a, *β*-catenin, and APC in the ALI group increased significantly, indicating that when ALI occurs in the body, the expression of Wnt5a, *β*-catenin, and APC can be upregulated. The occurrence and development of ALI may be related to the key point proteins of Wnt/*β*-catenin signaling pathway.

KGF-2 can combine with its receptors FGFR2-IIIb (KGFR) and FGFR1III-b to promote epithelial cell growth, differentiation, and migration [40]. In this study, KGF-2 was instilled into the lungs for pretreatment. Lung tissue injury in rats improved and permeability decreased, proving that KGF-2 has a protective effect on ALI induced by oleic acid in rats. Both leukocyte and erythrocytes were reduced in BALF of rats, and the expression of proinflammatory factors in the lung tissue homogenate was reduced, while the expression of anti-inflammatory factors was increased. It indicates that the protective effect of KGF-2 is related to reducing inflammatory response and improving inflammatory mediators. The expression of Wnt5a decreased after KGF-2 intervention, and *β*-catenin and APC decreased significantly. The results show that KGF-2 can downregulate *β*-catenin and APC protein expression, but its effect is not achieved by Wnt5a activation. The Wnt5a/*β*-catenin signaling pathway is just one of the Wnt/*β*-catenin signaling pathways. The expression of Wnt/*β*-catenin signaling pathway can also be achieved by Wnt/*β*-catenin signaling pathway initiated by Wnt3a and other nonclassical Wnt/*β*-catenin signaling pathways.

In this study, we analyzed the correlation between key proteins of Wnt/*β*-catenin signaling pathway, inflammatory factors, and the number of cells in BALF. The results further indicated that oleic acid-induced inflammation in rat ALI is related to the above proteins. The key proteins of the Wnt/*β*-catenin signaling pathway were preliminarily discussed, which laid a preliminary foundation for the next step to specifically study the mechanism of the Wnt/*β*-catenin signaling pathway in oleic acid-induced ALI.

KGF-2 is a protective drug, and there is no difference between the previous airway administration research centers and the control group. Therefore, this study only set up one intervention group, namely, ALI + KGF-2 group. The drug concentration of this group of pretreatment is 5 mg/kg, and it is administered 72 h in advance. This intervention method comes from the basis of previous research (see supplementary materials for details (available here)). The research on the key proteins of Wnt/*β*-catenin signaling pathway in this article is only a preliminary discussion on the key points in this pathway. It can only be initially proved that KGF-2 reduces the inflammatory reaction of ALI induced by oleic acid in rats, which may be related to the activation of key proteins in Wnt/*β*-catenin signaling pathway. The in-depth study on the Wnt/*β*-catenin signaling pathway including the use of Wnt/*β*-catenin signaling pathway activators and inhibitors will be further studied on this basis. In this study, the research on white blood cells was not classified. The presented results can only represent the overall leukocyte level and cannot reflect the specific leukocyte level. At the same time, the research on lung protection in this article only stays at the blood gas barrier stage, and the impact on alveolar epithelial cells and changes in alveolar surfactants cannot be reflected.

In summary, this study confirmed that KGF-2 pretreatment reduced the inflammatory response in ALI rats. The mechanism may be related to the activation of key proteins in the Wnt/*β*-catenin signaling pathway.

## Figures and Tables

**Table 1:** W/D ratio, LPI, and LIS of the three groups of rats.

Group	No. of animals	W/D ratio	LPI	LIS
Control group	15	4.51 ± 0.338	0.77 ± 0.050	0.94 ± 0.208
ALI group and	15	6.34 ± 0.247^*∗*^	3.25 ± 0.371^*∗*^	3.70 ± 0.330^*∗*^
ALI + KGF-2 group	15	5.54 ± 0.244^*∗∗*^	1.37 ± 0.271^*∗∗*^	2.46 ± 0.155^*∗∗*^

*Note.* KGF-2 is keratinocyte growth factor-2. ALI is acute lung injury. LIS is the ALI pathology score, the lung w/d ratio is the lung wet/dry weight (w/d) ratio, and LPI is the lung permeability index. Compared with control group, ^*∗*^*P* < 0.01; compared with ALI model group, ^*∗∗*^*P* < 0.01.

**Table 2:** Changes of TNF-*α*, IL-10, red blood cell, and white blood cell count of the three groups of rats.

Group	No. of animals	TNF-*α*	IL-10	Red blood cell count	White blood cell count
Control group	15	117.86 ± 77.20	1265.00 ± 496.31	3.6 ± 1.65	76.4 ± 9.50
ALI group and	15	1029.24 ± 173.61^*∗*^	224.42 ± 135.79^*∗*^	3638.3 ± 318.42^*∗*^	183.9 ± 11.45^*∗*^
ALI + KGF-2 group	15	531.48 ± 134.16^*∗∗*^	960.71 ± 310.96^*∗∗*^	2452.2 ± 255.49^*∗∗*^	159.2 ± 13.10^*∗∗*^

*Note.* TNF-*α* is tumor necrosis factor-*α*. IL-10 is interleukin-10. TNF-*α* and IL-10 were used to detect lung tissue homogenate, using enzyme-linked immunosorbent assay. Red blood cell and white blood cell count detection of bronchoalveolar lavage fluid. Compared with control group, ^*∗*^*P* < 0.01; compared with ALI model group, ^*∗∗*^*P* < 0.01.

**Table 3:** Correlation analysis between inflammatory reaction in ALI induced by oleic acid and expression of key proteins in Wnt/*β*-catenin signaling pathway.

Index		ALI group						ALI + KGF-2 group					
		Wnt5a		*β*-Catenin		APC		Wnt5a		*β*-Catenin		APC	
		*r*	*P*	*r*	*P*	*r*	*P*	*r*	*P*	*r*	*P*	*r*	*P*
Inflammatory factor	TNF-*α*	0.776	0.001	0.857	0.002	0.954	0.001	0.273	0.446	0.851	0.002	0.747	0.013
	IL-10	−0.879	0.001	−0.830	0.03	−0.697	0.025	−0673	0.033	−0.748	0.013	−0.852	0.002
Number of cells in BALF	White blood cell count	0.888	0.001	0.827	0.003	0.063	0.037	0.298	0.403	0.957	0.001	0.907	0.001
	Red blood cell count	0.794	0.006	0.721	0.019	0.879	0.001	0.588	0.074	0.875	0.001	0.772	0.009

*Note.* TNF-*α* is tumor necrosis factor-*α*. IL-10 is interleukin-10. Wnt5a, *β*-catenin, and APC are key proteins in the Wnt/*β*-catenin signaling pathway. BALF is bronchoalveolar lavage fluid.

## Data Availability

The data (figures and tables) used to support the results of this study can be obtained from the first author upon request.

## Supplementary Materials

Supplementary Materials Supplementary material is a study on the time and concentration of drug intervention.
