# Supplementary material for: Keratinocyte Growth Factor-2 Reduces Inflammatory Response to Acute Lung Injury Induced by Oleic Acid in Rats by Regulating Key Proteins of the Wnt/β-Catenin Signaling Pathway
Source: Evid Based Complement Alternat Med. 2020 Jun 20;2020:8350579. doi: 10.1155/2020/8350579 (PMC7322598; doi:10.1155/2020/8350579)

The protective effect of different concentrations of KGF-2 on oleic acid-induced acute lung injury in rats. When the concentration of KGF-2 was 5mg / kg, the pathological structure of lung tissue under light microscope was significantly better than that of KGF-2 at 2.5mg/kg. And there is no obvious difference with the lung tissue structure of KGF-2 concentration of 10mg/kg, so the drug concentration of 5mg/kg was selected in this study.

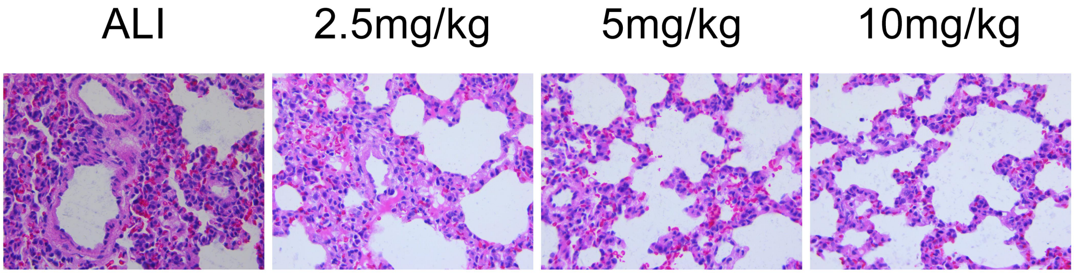

The pathological pictures of KGF-2 pretreated at different times are shown below. When KGF-2 pretreatment time was 72h, the pathological structure of lung tissue was significantly better than 60h and 84h. Therefore, the pretreatment time of this study is 72h.

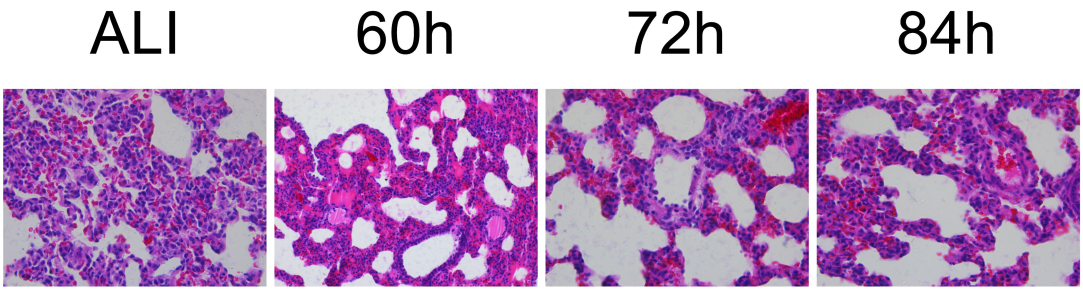

Supplement: Supplementary Materials — Supplementary material is a study on the time and concentration of drug intervention. [file 8350579.f1.pdf]
